# Arhythmia of the Heart

**Published:** 1905-03

**Authors:** 


**Arhythmia of the Heart.** By Dr. K. F. Wenckebach. Translated by Thos. Snowball, M.A., M.B. Pp. ix., 198. Edinburgh: William Green & Sons. 1904.

This is a very scientific work on irregularity of the heart. Commencing with a survey of the more recent views upon cardiac action, it later shows how clinical abnormalities of the rhythm of the action of the heart can be interpreted in the light of experimental research.

The writer first lays stress upon the intrinsic power of contraction of the heart—*i.e.* its myogenic action—and shows how the action can be altered by changes in stimulus production, excitability, stimulus conduction through the walls of the heart, and contractility.

The most common condition present in irregularity of the heart is the presence of what are known as "extra-systoles." These added systoles occur at varying lengths of time before the time at which the next normal systole should have occurred, and are followed by a pause longer than the ordinary diastole, a pause sufficiently long to bring the next normal systole into the place, so to speak, that it would have occupied if no extra-systole had occurred. In the simplest form of irregularity of the heart—*i.e.* an occasional intermission—this intermission is due to the occurrence of this extra-systole and the pause which follows it. On auscultating the heart, we are all familiar with the loud first sound which precedes the pause of an intermission and with the early occurrence of this loud first sound after the first sound which had gone before. The loud first sound is the sound which occurs during the extra-systole.

Into all the various questions of interest which are concerned in irregularity of the heart we cannot here enter, but the part played by interference with stimulus conduction, by altered excitability or contractility, are fully dealt with, as well as the part played by the nervous system in irregularities of the heart.

The writer more than once in his book mentions that questions concerning the action of the heart are not merely matters of scientific interest. He considers that much has still to be learnt, but that careful investigation and clinical observation, it is to be hoped, will enable us to ascertain accurately in any given case the cause of irregularity. Increased knowledge should not, however, aid diagnosis and prognosis only, but pharmacology in connection with various forms of cardiac irregularity needs also to be studied both experimentally and clinically.

